# Effects of muscle energy technique along conventional physical therapy after mesenchymal stem cell transplantation in knee osteoarthritis patients

**DOI:** 10.12669/pjms.40.11.9605

**Published:** 2024-12

**Authors:** Fanila Ashraf, Kinza Anwar, Hafsah Arshad

**Affiliations:** 1 Fanila Ashraf, MS-OMPT Railway General Hospital, Rawalpindi Islamabad, Pakistan; 2 Kinza Anwar, MS-OMPT Riphah International University, Islamabad, Pakistan; 3 Hafsah Arshad, MS-NMPT Ibadat International University, Islamabad, Pakistan

**Keywords:** Mesenchymal stem cells, Muscle energy technique, Conventional physical therapy, Rehabilitation protocol, Knee osteoarthritis

## Abstract

**Objective:**

To assess the efficacy of muscle energy technique (MET) in combination with conventional physical therapy compared to conventional physical therapy alone following mesenchymal stem cell transplantation in knee osteoarthritis (OA) patients.

**Methods:**

A randomized clinical trial was conducted at the Institute of Regenerative Medicine (IRM), Islamabad, Pakistan, for a time duration of 11 months from September 2022 to July 2023. Using non-probability purposive sampling, patients were randomly allocated to two treatment groups. Three treatment sessions per week for two weeks, lasting thirty minutes each, were administered with follow-up after one month. Numerical Pain Rating Scale, Western Ontario and McMaster Universities Arthritis Index, ROM, were used to assess the Pain, functional status, and Range of motion. Within Group-Analysis was done using Friedman with Wilcoxon signed-rank tests, while Mann-Whitney U-tests determined inter-group differences.

**Results:**

With-in Group-Analysis, showed statistically significant (p<0.05) at baseline, after one week, after two weeks, and after one month follow up in both the muscle energy technique (MET) Group-And conventional therapy group for NPRS, KNEE FLEXION ROM, and WOMAC. Between-Group-Analysis, also showed significant (p<0.05) for knee pain and WOMAC scores.

**Conclusions:**

Both groups are effective for improving knee pain, and functional limitation in knee osteoarthritis patients. However, MET Group along with conventional therapy showed marked effects on improving knee pain, knee flexion ROM, and functional limitations after mesenchymal stem cell transplantation in knee osteoarthritis patients.

## INTRODUCTION

Osteoarthritis (OA) is a progressive form of arthritis characterized by the inflammation and degradation of joint and cartilage. It stands out as the most prevalent among various joint diseases and is a leading cause of pain, disability, and functional limitations.^1^,^2^ Knee osteoarthritis (OA) is a chronic, degenerative metabolic bone disease with multiple distinct phenotypes, including the failure of chondrocytes to repair the articular cartilage.^3^,^4^ In individuals aged 40 and above, knee osteoarthritis emerges as the predominant factor causing impairments in functional mobility.^5^ Out of total worldwide population it is estimated that 10% males and about 13% females are affected by knee osteoarthritis, and osteoarthritis is considered as the fourth foremost cause of disability.^6^

Conventional treatment may provide clinical benefits, but it has limited role in repair of cartilaginous defects. Focus have been shifted to regenerative therapeutic procedures for the early treatment of cartilaginous defects.^7^ Various Regenerative procedure used to treat knee OA cartilage defect, the platelet rich plasma and Mesenchymal stem cells (MSCs) are harvested from the body of patient to be treated, to eliminate the risk of tissue or cell rejection from patient’s immune system.^8^ The ability of chondrogenic differentiation of MSCs is induced and supported by glycosaminoglycan and type-2 collagen which are components of cartilaginous matrix.^9^ MSCs have multidirectional differentiation potential and can be differentiated into osteoblasts and chondrocytes under specific induction conditions in vitro and in vivo, hence repairing bone and articular cartilage.^7^ MSCs possess immunosuppressive properties and are capable to reduce inflammation by suppressing T-cell activity and also influencing the pain associated with knee osteoarthritis.^10^

Rehabilitation plays a vital role to the success of regenerative medicine. Studies concluded that in response to exercise, articular chondrocytes increase their production of glycosaminoglycan’s and anti-inflammatory cytokines which leads to the improvements in cartilage’s organization and reduction in cartilage degeneration. It also highlighted that studies on rehabilitation strategies that can improve joint function following stem cell therapy are still relatively limited.^11^ Muscle energy technique (METs) basically enhances the muscle tolerance to stretching, which generates by the isometric contraction-induced activation of the Golgi tendon organs and decreases tension within muscles.

METs has been proven beneficial in reducing pain, inducing relaxation, and stimulating the body’s own healing mechanism in different procedures such as posterior Cruciate Ligament Reconstruction (PCLR).^12^ Hamstring muscles are considered to be principal stabilizers of the knee joint and Knee osteoarthritis can cause a decrease of muscle strength, range, and flexibility in the hamstring muscles.^13^ Exercise following bone marrow-derived mesenchymal stem cell therapy improves cartilage regeneration and skeletal health by decreasing the production of osteoclastogenic cytokines. Hence our study aimed to identify the additional effects of Muscle Energy Technique (MET) along with conventional physical therapy after mesenchymal stem cell transplantation in knee osteoarthritis regarding pain, range of motion, and functional disability.

## METHODS

A randomized clinical trial (registered with clinical trials with registry no. (NCT05349565) was conducted at the Institute of Regenerative Medicine (IRM), Islamabad, Pakistan, for a time duration of 11 months i.e. from September 2022 to July 2023.

### Participants:

A total of 30 participants were assessed for eligibility, who visited IRM during the recruitment period. However, 26 participants fulfilled the inclusion criteria and showed a willingness to participate in the study and were thus recruited through non-probability Purposive sampling technique. The participants were randomly divided into two treatment groups i.e. Muscle energy technique group (n=13), conventional therapy group (n=13).

### Ethical Approval:

The study was initiated after the approval from the Internal Review Board of Riphah College of Rehabilitation and Allied Health Sciences with Ref # Riphah/RCRS/REC 01255, Dated: August 11, 2022.

### Inclusion & Exclusion Criteria:

The inclusion criteria were both genders male and female, Age > 40 years, bilateral knee Osteoarthritis, 11^th^ post op day of patient status post mesenchymal stem cell transplantation. Patient with History of recent or past septic arthritis, Unilateral knee Osteoarthritis, Patients with neurological deficit, uncontrolled diabetes, Malignancy, Vascular insufficiency, Knee osteoarthritis patients undergone mesenchymal stem cell transplantation were excluded from the study.

### Randomization:

Randomization of the participants was done through the sealed envelope method by using a computerized random number generator. The sequence of random allocation was done by an individual who was not directly involved in the study. Consecutive random numbers were written on index cards and placed in thick and opaque sealed envelopes before the study. After taking consent from the patient for their participation in the study, the treating physiotherapist opened the envelope and gave the respective treatment to the patient. The study was single blinded as the participants were not aware of the interventional group they were placed in.

### Intervention:

The MET group received MET alongside conventional therapy, while the control group received conventional therapy (CT) only. Both groups received a 10 minutes application of high-frequency TENS applied to knee joint prior to their respective sessions. In MET GROUP, Muscle energy technique was applied on hamstring muscle while engaging the first barrier then Isometrically hamstring were contracted against 20% body’s resistance (6-10 seconds hold). Prompt relaxation, followed by gentle stretching during post-isometric relaxation. Breathing and eye movements were utilized. This process is repeated to incrementally enhance the range of motion (4-5 reps). This Group-Also received conventional physical therapy as described below: Conventional physical therapy group received Ankle pumps, Heel slides, Open chain knee flexion and extension exercises, Straight leg raise, Quad isometrics and Calf stretch. All these exercises were given 10 repetitions x1 set, three days/week.

A 30 minutes session was given to each patient and three sessions per week were given for two weeks and follow up data was collected after one month. Baseline assessment was done after the patients were enrolled in the research and re-assessment was done after 1st week, 2^nd^ week and then after one month in which outcome measures of pain, range of motion, and functional status were assessed.

### Outcome Measures:

The Numeric Pain Rating Scale (NPRS) is the simplest and most commonly used numeric scale to rate the pain from 0 (no pain) to 10 (worst pain). The NPRS was used for subjective pain measurement that has good test-retest reliability.^14^ A goniometer is an instrument that measures the available range of motion at a joint. In this study, knee ROMS was measured by goniometer before and after the exercise program.^15^ The Western Ontario and McMaster Universities Osteoarthritis Index (WOMAC) was used to measure symptoms and physical disability, originally developed for people with OA of the hip or knee. It evaluates three dimensions: pain, stiffness, and physical function.^16^

### Statistical Analysis:

The normality of the data was assessed by using the Shapiro Wilk test and the data was non-normally distributed (p<0.005) for pain, ROM, and WOMAC score, thus non-parametric were applied. Within Group-Analysis was done using Friedman along Wilcoxon signed rank test as Wilcoxon signed rank test is used for week wise comparison of data and is interpreted in the form of p-values. For between Group-Analyses Mann Whitney U-test was applied. As the data was quantitative non-parametric, we represented it as a median and interquartile range, from quartile 1 (25%) to quartile 3 (75%). All data were analyzed using SPSS 28.0, considering a two-tailed level of p<0.005 to be statistically significant for analysis.

## RESULTS

Out of 26 participants, 13 participants were randomly assigned into Treatment Group-A (MET) and 13 participants were included in Treatment Group-B (Conventional therapy group). 11 (42.3%) were males and 15 (57.7%) were females. Mean age of participants in Treatment Group-A (MET) was 63.46±9.28 and mean age of participants in Treatment Group-B (Conventional therapy group) was 65.23±8.6.

With-in Group-Analysis, Friedman T-test with Wilcoxon was applied for pain, ROM, and WOMAC score. NPRS showed significant improvements (p<0.05) at baseline, after one week, after two weeks, and after one month follow up in both the muscle energy technique (MET) Group-And conventional therapy group. Right and left side Knee flexion showed significant improvement from the baseline till the follow up in both groups (p<0.05). Right knee Extension in MET Group-Also showed significant improvement at follow up (p<0.05). But Left-side Knee extension showed non-significant improvement from the baseline till the follow up in both groups (p>0.05). As shown in Table-I. Total WOMAC score showed significant improvement from the baseline till the follow up in both groups (p<0.05).

**Table-I:** Friedman T-test with Wilcoxon test –NPRS and ROM.

		MET group				CT group			

		Median (IQR)	MR	Z (df)	p-value	Median (IQR)	MR	Z (df)	p-value
NPRS	Baseline	7.00 (1)	4.00	-3.314^a^	<.001***	7.00 (1.5)	3.69	-2.236^a^	.025*
Rom Knee Extension Right	Baseline	180 (5.5)	1.85	-2.236^a^	.025	180 (5)	2.04	-1.826^a^	.068
	After 1^st^ Week	180 (3.5)	2.31	-2.032^b^	.042	180 (3.5)	2.42	-1.604^b^	.109
	After 2^nd^ Week	180 (2)	2.77	-1.826^c^	.068	180 (2)	2.65	-1.633^c^	.102
	After 1 Month	180 (0)	3.08	16.333^d^(3)	.027*	180 (0)	2.88	11.000^d^(3)	.068
Rom Knee Extension Left	Baseline	180 (4.5)	2.04	-1.890^a^	0.59	180 (0)	2.27	-1.414^a^	.157
	After 1^st^ Week	180 (1.5)	2.35	-1.826^b^	0.68	180 (0)	2.42	-1.342^b^	.180
	After 2^nd^ Week	180 (0)	2.77	-1.000^c^	.317	80 (0)	2.58	-1.414^c^	.157
	After 1 Month	180.00 (0)	2.85	11.757^d^(3)	.066	180 (0)	2.73	6.000^d^(3)	.180

Significance level: p<0.0e01***, p<0.05*,

a =baseline and 1^st^ week comparison,

b =1^st^ week and 2^nd^ week comparison,

c =2^nd^ week and 1 month comparison, d=baseline and 1 month comparison.

In the MET group, median value reduced from 62 at the baseline to 32 at the follow up and is significant (p<0.001) whereas, for the conventional therapy group, median score reduced from 65 at the baseline to 47 at the follow up and is significant (p<0.001) with large effect size from baseline to follow up in both groups. Hence better results are observed in the MET group in Table-II. Between-Group-Analysis, was done by Mann Whitney U-test. For knee pain showed significant improvement (p<0.05) in both the MET Group-And the conventional therapy group. Right & left side Knee extension and flexion showed non-significant improvement from the baseline till the follow up in both groups (p>0.05). According to median (IQR) score better results are observed in the MET group for NPRS and range of motion, presented in Table-III. Total WOMAC score showed significant improvement from the baseline till the follow up in both groups (p<0.05). According to median (IQR) score better results are observed in the MET group for pain, physical function and overall WOMAC scoring Table-IV.

**Table-II:** Friedman T-test with Wilcoxon test –WOMAC.

WOMAC		MET group				CT group			

		Median (IQR)	MR	Z(df)	p-value	Median (M)	MR	Z (df)	p-value
severity of pain	Baseline	12 (4.5)	3.85	-2.810^a^	.005	14 (1)	3.73	-2.449^a^	.0148*
	After 1^st^Week	11 (3)	3.15	-3.228^b^	.001	14 (1)	3.23	-3.082^b^	.002
	After 2^nd^ Week	7 (4.5)	1.65	-1.730^c^	.084	12 (3)	1.88	-2.719^c^	.007
	After 1 Month	6 (3.5)	1.35	36.529^d^(3)	<.001***	10 (4)	1.15	36.432^d^(3)	<.001***
Stiffness	Baseline	6 (1)	3.85	-2.887^a^	.004**	5 (2)	3.81	-2.640^a^	.008
	After 1^st^ Week	5 (2)	3.12	-3.153^b^	.002**	5 (2)	3.12	-3.127^b^	.002
	After 2^nd^ Week	4 (1)	1.92	-3.162^c^	.002**	3 (1)	1.88	-2.828^c^	.005
	After 1 Month	3 (1)	1.12	36.910^d^(3)	<.001***	2 (2)	1.19	35.898^d^(3)	<.001***
Level Of Difficulty	Baseline	44 (3.5)	4.00	-3.246^a^	.001	46 (4)	4.00	-3.189^a^	.001
	After 1^st^ Week	43 (8)	3.00	-3.210^b^	.001	44 (7)	3.00	-3.185^b^	.001
	After 2^nd^ Week	27 (2)	2.00	-3.240^c^	.001**	36 (12)	1.92	-3.096^c^	.002**
	After 1 Month	23 (2)	1.00	39.000^d^(3)	.001**	32 (9.5)	1.08	37.892^d^(3)	.001**
Total Womac Score	Baseline	62 (8)	4.00	-3.203^a^	.001**	65 (7.5)	4.00	-3.187^a^	.001**
	After 1^st^ Week	59 (13)	3.00	-3.190^b^	.001**	63 (9)	3.00	-3.186^b^	.001**
	After 2^nd^ Week	38 (7.5)	2.00	-3.205^c^	.001**	51 (15.5)	2.00	-3.187^c^	.001**
	After 1 Month	32 (7)	1.00	39.000^d^(3)	.001***	47 (12)	1.00	39.000^d^(3)	.001***

**Table-III:** Mann Whitney U -test- NPRS and ROM.

Variables	No of weeks	MET	CT	Median	IQR	P-value

		MR	MR			
NPRS	Baseline	12.12	14.88	7.00	1	.322
	After 1^st^ Week	8.54	18.46	6.00	2	<0.001
	After 2^nd^ Week	7.42	19.58	4.50	2.25	<0.001
	After 1 Month	7.69	19.31	4.50	3	<0.001***
Rom Knee Flexion Right	Baseline	13.08	13.92	123.00	19.25	.777
	After 1^st^ Week	13.46	13.54	126.00	17.5	.979
	After 2^nd^ Week	13.58	13.42	130.00	15.75	.959
	After 1 Month	13.96	13.04	133.00	9.75	.756
Rom Knee Flexion Left	Baseline	11.92	15.08	126.00	20	.288
	After 1^st^ Week	12.15	14.85	129.00	18	.365
	After 2^nd^ Week	12.23	14.77	133.00	17	.383
	After 1 Month	12.81	14.19	135.00	13.25	.630
Rom Knee Extension Right	Baseline	12.73	14.27	180.00	4.75	.558
	After 1^st^ Week	12.65	14.35	180.00	2.75	.490
	After 2^nd^ Week	13.08	13.92	180.00	1.5	.718
	After 1 Month	13.0	14	180.00	0	.547
Rom Knee Extension Left	Baseline	12.58	14.42	180.00	0.75	.404
	After 1^st^ Week	12.65	14.35	180.00	0.25	.445
	After 2^nd^ Week	14.8	12.92	180.00	0	.448
	After 1 Month	13.5	13.5	180.00	0	1.0

**Table-IV:** Mann Whitney U-test for WOMAC.

Variables	No of weeks	MET	CT	Median	IQR	P-value

		MR	MR			
Severity of Pain	Baseline	8.73	18.27	14.00	2	<.001
	After 1^st^ Week	8.08	18.92	13.00	3	<.001
	After 2^nd^ Week	7.23	19.77	9.00	5.25	<.001
	After 1 Month	7.88	19.12	9.00	4.25	<.001***
Stiffness	Baseline	13.42	13.58	5.00	1	.955
	After 1^st^ Week	12.85	14.15	5.00	2	.649
	After 2^nd^ Week	14.19	12.18	3.00	1	.601
	After 1 Month	13.38	13.62	2.50	1.25	.960
Level of Dificulty	Baseline	9.12	17.88	45.00	2	.002
	After 1^st^ Week	10.62	16.38	43.00	8	.057
	After 2^nd^ Week	9.19	17.81	27.00	9.5	.003
	After 1 Month	8.38	18.62	24.00	9.5	<.001***
Total Womac Score	Baseline	9.35	17.65	63.00	4.25	.004
	After 1^st^ Week	9.85	17.15	60.00	12.25	.014
	After 2^nd^ Week	8.15	18.85	39.50	14	<.001
	After 1 Month	8.27	18.73	36.00	15.5	<.001***

## DISCUSSION

The current study showcased significant differences between the muscle energy technique (MET) and conventional therapy groups. Patients undergoing MET after mesenchymal stem cell transplantation reported noticeable improvements in pain, range of motion, and WOMAC scores. MET consistently demonstrated notable enhancements in pain levels, knee range of motion, and functional outcomes till the follow-up period.

Shinde S, evaluated the effects of muscle energy technique and conventional approach on the functional outcomes of those patients who went through total knee arthroplasty. Better outcomes were documented in the METs Group-As compared to conventional exercises alone on all of the three components of WOMAC scale, ROM and muscle strength in total knee arthroplasty (TKA) rehabilitation.^17^ The results of this study are consistent with the current study in which more pronounced improvements in pain reduction and overall WOMAC scores were noted in MET group as compared to conventional therapy, highlighting its potential as an important treatment modality for knee osteoarthritis patient’s post-mesenchymal stem cell transplantation. Pain reduction could be attributed by MET’s hypoalgesic effects in response to the inhibitory Golgi tendon reflex, which is induced during isometric contraction and leads to muscle reflex relaxation.^18^,^19^

A case report by the Justin Het describes the six weeks outpatient physical therapy management of 65 years old male with chief complaint of bilateral knee pain status post stem cell cartilage replacement therapy. Conventional physical therapy protocol was given to patient for six weeks, significant improvement in strength, moderate increases in ROM, and moderate decreases in pain was recorded after following the protocol, along with improved functional ambulation endurance.^20^ These findings were consistent with the Current study in which additional effect of METs was observed along with conventional therapy, and within Group-Analysis showed significant improvement in pain, ROM and functional limitations.

Exercise therapy provides colossal effects on stem cell by increasing the production of glycosaminoglycan’s and anti-inflammatory cytokines, which leads to the improvements in cartilage’s organization and reduction in cartilage degeneration which ultimately improves physical function.^21^,^22^ Eileen Danaher Hacker et.al conducted a RCT to evaluate the effect of strength training after hematopoietic stem cell therapy (HCT). Study proved that six weeks strength training protocol improves early recovery after HCT, and helps in reduction of fatigue, preserving muscular strength and function ability. This study also emphasized on the importance of balanced exercise treatment interventions following HCT, making it robust enough to alter outcomes in chronic illness or critical ill patients.^23^ The results are consistent with the current study, which also measured the functional outcomes such as knee stiffness, and physical function, and a positive outcome was observed that showed an overall improvement in the knee functional status of the enrolled subjects.

According to Sophie Ruth Allen and Anthony Wright’s review, there are substantial post-operative rehabilitation regimens for other techniques of cartilage restoration such as ACL and micro fracture/micro drilling, whereas stem cell therapy currently lacking established physical therapy protocols. However, a meta-analysis showed that rehabilitation was a significant outcome modifier of MSC treatment, indicating the potential importance of an appropriate rehabilitation approach.^24^ Similarly another study was conducted by Gilani SS et.al on use of platelet rich plasma (PRP) along with exercise therapy in knee OA treatment which concluded that PRP combined with exercises is found to be extremely effective treatment for pain relief and functional status of Grade-III knee OA.^25^ These findings are also in line with recent study in which intervention including METs along with conventional physical therapy have significant improvement in pain, and functional limitations at each level of assessment till the follow up session in knee OA patients.

McKay J et al. concluded that MSC recipients are an understudied group, despite being one of the most vulnerable to physical deconditioning after transplantation. The study also found that rehabilitation following regenerative therapy yields promising treatment outcomes. However, additional research must be undertaken to provide clinical evidence.^26^ Their findings also support the current trail. To our knowledge, current study is the first study that incorporated a muscle energy technique, that is manual therapy approach to conventional therapy in post MSC patient and showed METs along conventional therapy has positive effects on knee OA regarding pain, ROM, and functional capacity.

### Limitation:

It includes short duration, single center study that may affect the generalizability of data. The number of male and female participants were also not equal. Post regenerative rehabilitation is more challenging, proper well documented rehabilitation protocol is required. Post-stem cell therapy patients should receive precise, long-term rehabilitation approaches that incorporate various soft tissue procedures along EMG investigations, with a six months follow-up.

## CONCLUSION

The current study concluded that the muscle energy technique in conjunction with conventional treatment and conventional physical therapy alone, are effective treatments for improving knee pain, and functional limitations. Notably, MET Group-Along conventional therapy showed greater efficacy in improving knee pain, knee ROM, and functional improvement, highlighting its potential as a valuable adjunctive treatment modality in knee OA rehabilitation post-MSC transplantation.

### Authors’ Contributions:

**FA:** Acquisition of data, Analysis and interpretation of data. Drafting the article and revising it critically for important intellectual content.

**KA: C**onception and design, Data analysis and interpretation. Critical review and revision. Final approval of the version to be published. Accountable for clinical integrity of the study.

**HA:** Drafting the article and revising it critically.

## References

[1] Wang Q, Runhaar J, Kloppenburg M, Boers M, Bijlsma JW, Bierma-Zeinstra SM (2021). Diagnosis of early stage knee osteoarthritis based on early clinical course:data from the check cohort. Arthritis Res Ther.

[2] Al-Khlaifat L, Okasheh R, Muhaidat J, Hawamdeh ZM, Qutishat D, Al-Yahya E (2020). Knowledge of knee osteoarthritis and its impact on health in the Middle East:are they different to countries in the developed world?A qualitative study. Rehabil Res Pract.

[3] Krishnasamy P, Hall M, Robbins SR (2018). The role of skeletal muscle in the pathophysiology and management of knee osteoarthritis. Rheumatology.

[4] Vitaloni M, Botto-van Bemden A, Sciortino Contreras RM, Scotton D, Bibas M, Quintero M (2019). Global management of patients with knee osteoarthritis begins with quality of life assessment:a systematic review. BMC Musculoskelet Disord.

[5] Masekar MB, Rayjade DA, Yadav DT, Chotai DK (2021). Effectiveness of muscle energy technique and proprioceptive neuromuscular facilitation in knee osteoarthritis. Int J Life Sci Pharma Res.

[6] Huang R, Li W, Zhao Y, Yang F, Xu M (2020). Clinical efficacy and safety of stem cell therapy for knee osteoarthritis:A meta-analysis. Medicine.

[7] Freitag J, Bates D, Wickham J, Shah K, Huguenin L, Tenen A (2019). Adipose-derived mesenchymal stem cell therapy in the treatment of knee osteoarthritis:a randomized controlled trial. Regene Med.

[8] Boric I, Hudetz D, Rod E, Jelec Z, Vrdoljak T, Skelin A (2019). A 24-month follow-up study of the effect of intra-articular injection of autologous microfragmented fat tissue on proteoglycan synthesis in patients with knee osteoarthritis. Genes.

[9] Kalamegam G, Memic A, Budd E, Abbas M, Mobasheri A (2018). A comprehensive review of stem cells for cartilage regeneration in osteoarthritis. Adv Exp Med Biol.

[10] Lukomska B, Stanaszek L, Zuba-Surma E, Legosz P, Sarzynska S, Drela K (2019). Challenges and controversies in human mesenchymal stem cell therapy. Stem Cells Int.

[11] Smith JK (2020). Exercise as an adjuvant to cartilage regeneration therapy. Int J Mol Sci.

[12] Sobariyah AM, Efendi EN, Perdana SS (2022). The Effect Of Muscle Energy Technique (MET) on Range of Motion (ROM) Knee Joint in Patients Postoperative Posterior Cruciate Ligament Reconstruction (PCLR) Hamstring Graft:A Case Study. Academic Physiother Conference Proc.

[13] Anjum N, Sheikh RK, Omer A, Anwar K, Khan MMH, Aftab A (2023). Comparison of instrument-assisted soft tissue mobilization and proprioceptive neuromuscular stretching on hamstring flexibility in patients with knee osteoarthritis. PeerJ.

[14] Greenwood R, Ellison J, Gleeson P, Mitchell K (2022). Reliability of pain scores during a body weight support protocol in individuals with knee osteoarthritis. Disability Rehabil.

[15] Epskamp S, Dibley H, Ray E, Bond N, White J, Wilkinson A (2020). Range of motion as an outcome measure for knee osteoarthritis interventions in clinical trials:an integrated review. Physical Ther Rev.

[16] Salaffi F, Leardini G, Canesi B, Mannoni A, Fioravanti A, Caporali Ro (2003). Reliability and validity of the Western Ontario and McMaster Universities (WOMAC) Osteoarthritis Index in Italian patients with osteoarthritis of the knee. Osteoarthritis Cartilage.

[17] Shinde S (2020). Effect of muscle energy technique and conventional approach on functional outcomes in total knee arthroplasty-a comparative study. Int J Psychosocial Rehabil.

[18] Ahmed K, Batool A, Zahid H, Bashir U, Ali M (2023). Comparing the Therapeutic Effects of Dry Needling and Muscle Energy Technique on Quadratus Lumborum Trigger Points:A Study on Back Pain Alleviation. J Health Rehabil Res.

[19] Kang Y-H, Ha W-B, Geum J-H, Woo H, Han Y-H, Park S-H (2023). Effect of muscle energy technique on hamstring flexibility:systematic review and meta-analysis. Healthcare (Basel).

[20] Hett J (2019). Outpatient PT Management of Patient with Bilateral Knee Pain S/P Stem Cell Cartilage Replacement Therapy of Both Knees. https://commons.und.edu/pt-grad/670/.

[21] Zeng C-Y, Zhang Z-R, Tang Z-M, Hua F-Z (2021). Benefits and mechanisms of exercise training for knee osteoarthritis. Front Physiol.

[22] De Sire A, Marotta N, Marinaro C, Curci C, Invernizzi M, Ammendolia A (2021). Role of physical exercise and nutraceuticals in modulating molecular pathways of osteoarthritis. Int J Mol Sci.

[23] Hacker ED, Collins E, Park C, Peters T, Patel P, Rondelli D (2017). Strength training to enhance early recovery after hematopoietic stem cell transplantation. Biol Blood Marrow Transplant.

[24] Allen SR, Wright A (2019). Stem cell therapy for knee osteoarthritis:A narrative review of a rapidly evolving treatment with implications for physical therapy management. Phys Ther Rev.

[25] Gilani SS, Naeem M, Bukhari T (2017). Use of platelet rich plasma with exercisesin the treatment. Rawal Med J.

[26] McKay J, Nasb M, Hafsi K (2022). Mechanobiology-based physical therapy and rehabilitation after orthobiologic interventions:a narrative review. Int Orthop.

